# Early Life Nutrition and Energy Balance Disorders in Offspring in Later Life

**DOI:** 10.3390/nu7095384

**Published:** 2015-09-21

**Authors:** Clare M. Reynolds, Clint Gray, Minglan Li, Stephanie A. Segovia, Mark H. Vickers

**Affiliations:** Liggins Institute and Gravida: National Centre for Growth and Development, University of Auckland, Auckland 1142, New Zealand; E-Mails: c.reynolds@auckland.ac.nz (C.M.R.); c.gray@auckland.ac.nz (C.G.); m.li@auckland.ac.nz (M.L.); s.segovia@auckland.ac.nz (S.A.S.)

**Keywords:** developmental programming, maternal nutrition, energy balance, metabolic syndrome

## Abstract

The global pandemic of obesity and type 2 diabetes is often causally linked to changes in diet and lifestyle; namely increased intake of calorically dense foods and concomitant reductions in physical activity. Epidemiological studies in humans and controlled animal intervention studies have now shown that nutritional programming in early periods of life is a phenomenon that affects metabolic and physiological functions throughout life. This link is conceptualised as the developmental programming hypothesis whereby environmental influences during critical periods of developmental plasticity can elicit lifelong effects on the health and well-being of the offspring. The mechanisms by which early environmental insults can have long-term effects on offspring remain poorly defined. However there is evidence from intervention studies which indicate altered wiring of the hypothalamic circuits that regulate energy balance and epigenetic effects including altered DNA methylation of key adipokines including leptin. Studies that elucidate the mechanisms behind these associations will have a positive impact on the health of future populations and adopting a life course perspective will allow identification of phenotype and markers of risk earlier, with the possibility of nutritional and other lifestyle interventions that have obvious implications for prevention of non-communicable diseases.

## 1. Background

The prevalence of overweight and obesity has risen markedly in Western societies over the past few decades and the trend is mirrored in developing nations that are transitioning to first-world economies. Obesity results from a chronic imbalance between energy intake and energy expenditure [1]. As such, disorders of systems regulating energy balance can lead to obesity and related metabolic complications. In this context, it is now well established that alterations in the early life nutritional environment can lead to perturbations in the regulation of energy balance and energy sensing pathways in later life. The Developmental Origins of Health and Disease (DOHaD) hypothesis has highlighted the link between the periconceptual, fetal, and early infant phases of life and the subsequent development of metabolic disorders in later life [2,3,4]. Under the DOHaD model, the fetus or neonate makes predictive adaptations in response to cues from the early life environment, resulting in adjustments in homeostatic systems to aid immediate survival and improve success in an expected postnatal environment of adversity. However, inappropriate interpretations of these cues or changes to the environment may result in a mismatch between prenatal predictions and postnatal reality. As a result, these adaptations, or predictive adaptive responses (PARs) [5], may be disadvantageous in postnatal life, leading to an increased risk of chronic diseases in adulthood that may be transmitted into future generations [6]. In particular, the hypothalamic neuronal circuits located in the arcuate nucleus controlling appetite and energy expenditure are set early in life and have been shown in a number of models to be perturbed by a range of early life nutritional insults [7]. These include epigenetic modifications which regulate hypothalamic gene expression and therefore represent potential molecular mechanisms linking maternal diet during pregnancy to the later risk of disorders in energy balance and obesity in offspring. Disorders of energy balance as a consequence of developmental programming remain poorly defined on a mechanistic basis. Human evidence is limited and based largely on semi-quantitative approaches or limited metabolic physiological endpoints. Animal models have provided some mechanistic insight but this has primarily focused around hypothalamic re-programming and integration with behavioral/food preferences and direct measures of energy expenditure still remain largely unexplored.

## 2. Developmental Programming and Disorders of Energy Balance: Human Evidence

Evidence from both epidemiological studies and intervention studies in animal models has shown that the metabolic programming of energy balance begins with and can be modified by nutrition in the very early stages of development. Therefore, pregnancy and lactation are revealed as critical periods where food restriction may lead to permanent adaptations with lasting effects on metabolic mechanisms in the offspring; thereby changing the predisposition to obesity in adult life. Evidence to date has shown that the different outcomes of these adaptations on later health depend on the type, duration, period, and severity of the restriction and that they are, at least in part, sex specific.

The Dutch Famine (1944–1945) cohort is one of the most cited human cohorts used to examine the effects of poor early life nutrition on later metabolic and cardiovascular outcomes. Individuals exposed to the famine have increased adiposity, which may arise due to changes in energy intake, physical activity, and/or metabolic efficiency and are also dependent upon the timing of famine exposure [8,9]. Many of these data are collated via food frequency and physical activity data questionnaires and associations appear to differ according to the reference population used [10]. However, persistent small changes in energy balance could explain the increased weight of famine-exposed individuals. Further, work by Lussana *et al.* reported that famine exposure was associated with a preference for fatty foods and a tendency to be less physically active when studied in men and women at 58 years of age [11]. Data from adults born between 1954 and 1964 from the 2002 China National Nutrition and Health Survey show that exposure to the Chinese famine during fetal life or infancy was associated with an increased risk of metabolic syndrome in adulthood but little is known about altered regulation of energy balance in this cohort [12]. In the Helsinki Birth Cohort Study, with participants aged 56–70, whose birth weight and length were recorded, it was concluded that prenatal growth may modify food and macronutrient intake later in life, and altered dietary habits could potentially explain the increased risk of chronic disease in individuals born with small body size [13]. There are also reports on the effects of seasonality on fetal growth and postnatal outcomes in offspring related to alterations in energy balance. For example, in rural Gambian women, pronounced naturally occurring seasonal differences in diet can lead to persistent and systemic changes in epigenetic profiles in offspring including altered methylation at imprinted loci [14,15].

Recent work by Neymotin and Nemzer has also detailed a concept around “locus of control” as relates to obesity [16]. Health locus of control has been associated with a variety of health outcomes and designed to predict behaviors and cognitive processes relevant to both mental and physical health [17,18]. In the context of energy balance, this concept suggests that, in humans, overlaid on the complex system for modulating energy intake, there exists a wide variation in “perceived locus of control”. Locus of control is an important characteristic in relation to obesity as, by definition, it indicates whether an individual believes that his or her environment and choices are under his or her control. An example of this is the treatment of obese individuals with dexamphetamine (for a psychotropic effect on motivation) coupled with a basic behavioural intervention which was shown to be effective for weight loss with no significant complications reported [19]. Further work by Levy *et al.* has used stimulant medication (mainly amphetamine) in a female cohort of obese individuals with attention deficit hyperactivity disorders; significant weight lost was observed and suggested to be a result of positive effects of treatment on self-directedness, persistence and behavioural change as regards diet compliance [20]. Thus, in addition to actual physical cues of hunger or satiation, the ability to interpret those cues appropriately in a given social setting may help determine how obesity develops and persists and therefore may be a component underlying developmental programming of altered energy balance that has yet to be explored.

## 3. Developmental Programming and Disorders of Energy Balance: Evidence from Experimental Models

Human evidence is limited due to long generation times for prospective studies and the quality of data records for retrospective studies. Data are primarily derived from rodent models due to the short timeframe required to generate offspring and there are now a number of examples showing lasting effects of the perinatal environment on neurodevelopment and regulation of energy balance [21,22,23]. Circulating hormones influence multiple aspects of hypothalamic development and play a role in directing formation of neural circuits. Thus, a deficiency in any of these essential factors during a critical period of development may have permanent structural consequences. Hypothalamic development in large mammals is in contrast to the situation in rodents that are either altricial at birth or born immature [24]. Consequently, hypothalamic maturation in the rodent is initiated prenatally but does not finalise until the second week of postnatal life [25]. Nutrient supply is therefore determined by maternal lactational capacity rather than being dependent on placental nutrient transfer capacity. Changes in milk composition therefore have the potential to have a pronounced effect on extent of hypothalamic maturation mediated through changes in hormone concentration of the milk. For example, rat pups born to obese dams show a precocious surge in circulating insulin together with an abnormal and pronged neonatal leptin surge [26]. These effects are likely to be driven by changes in milk composition. As an example, a maternal high fat diet in the rat is associated with increased fat [27,28] and protein concentrations in milk which appears to induce early overnutrition and has been shown to be paralleled by obesity and adrenal and thyroid dysfunction in male offspring at the time of weaning [29]. However, despite the different developmental time windows across the range of models used, both altricial and precocial species share several common offspring programming mechanisms, particularly as regards hypothalamic re-wiring.

A particular focus has been on the adipokine leptin, an important neurotropic factor during postnatal development [30,31]. Leptin deficiency during critical periods of development may have later consequences in body weight regulation and control of appetite and energy expenditure [32,33]. In the rat, pre- and postnatal calorie restriction perturbs hypothalamic neuropeptide regulation of energy balance, altered by the timing and magnitude of the leptin surge and therefore setting the stage for hyperphagia and reduced energy expenditure, hallmarks of obesity. Leptin in turn reverses this phenotype by increasing hypothalamic ObRb signaling (sensitivity) and affecting only the orexigenic arm of the neuropeptide balance [34]. Importantly, leptin replacement in the neonatal period has been shown in some experimental animal models to rescue the effects associated with adverse early life developmental programming [34,35,36,37]. However, the effects of leptin can differ based on the experimental model used. For example leptin intervention appears directionally (and sex) dependent upon the level of maternal nutrition [38]—it can be protective in offspring of mothers undernourished during pregnancy [35,37] but may induce an adverse phenotype when treatment is provided to offspring of normally nourished mothers [36,39].

The brain is not the only tissue that is sensitive to perinatal conditions and responsible for the perinatal programming of body weight control. Recent data indicate that the development of other structures, such as peripheral innervations of the sympathetic nervous system, may also be altered during development and have an important impact on future adiposity and metabolism of the offspring [40]. In addition, an altered early life environment may modify food and taste preferences in offspring [41]. As an example, prenatal to a maternal low protein (LP) diet programmes a preference for high fat foods in young adult offspring [42]. Such changes may be driven by alterations in taste receptor expression. Recent studies have demonstrated that taste receptors in tongue, gut, and pancreas are associated with local hormone secretion including that of TNF-α and leptin [43]. In these studies, in addition to the regulation of food intake, taste perception appeared to be tightly linked to circulating metabolic hormone levels. Further understanding of the link between taste perception and peripheral metabolic control could therefore potentially lead to the development of intervention therapies for obesity or type 2 diabetes (T2DM).

Studies in the rodent have primarily utilised maternal undernutrition (primarily either global or LP) or uterine artery ligation to induce intrauterine growth restricted (IUGR) offspring. In addition, it is also proposed that intrauterine stress exposure may interact with the nutritional milieu, and that stress biology may represent an underlying mechanism mediating the effects of diverse intrauterine perturbations and later programming effects on brain and peripheral targets of programming of body composition, energy balance homeostasis and metabolic function [44]. Several studies utilising maternal perinatal undernutrition have produced consistent evidence that the programmed offspring present with low birth weight, hyperphagia in postnatal life and develop obesity, insulin resistance and sedentary behaviours in adulthood [31,45,46,47]. The hyperphagia is exacerbated in the presence of a postnatal high fat diet (HFD) [45]. In addition to structural changes in the hypothalamus, the rodent models have also shown modifications in key neuropeptides in the arcuate nucleus including the anorexigenic proopiomelanocortin (POMC), and the orexogenic peptides agouti gene-related protein (AgRP) and neuropeptide Y (NPY). Although the timing and severity of undernutrition differs across models, the data appear consistent with increased hypothalamic NPY and AgRP and decreased POMC gene expression in postnatal offspring [7,48,49]. In addition to changes in AgRP, offspring from malnourished dams also present with dysregulation in the hypothalamic melanocortin (melanocortin receptor 4) and α-melanin-stimulating hormone (α-MSH) system with a series of alterations: impaired neurogenesis and neuronal functionality, disorganization of feeding pathways, impaired glucose control, and leptin/insulin resistance. Overall, these alterations may account for the long-lasting dysregulation of energy balance and obesity [50]. In addition to leptin, the impact of maternal UN on other factors such as serotonin (5-HT) and dopamine and later disorders of energy balance have also been investigated [51]. In the rodent, maternal undernutrition is associated with changes in hypothalamic 5-HT and dopamine expression concomitant with hyperphagia and obesity.

Although the rodent has a number of advantages over other models species (including short gestation length and relative ease of genetic manipulation), the critical periods for the setting of pathways regulating energy balance differ significantly between rodents and humans [52]. The setting down of neural pathways develops in the early postnatal period in the rodent while in the human and other model species (e.g., sheep, primate, pig) these pathways develop prenatally [53]. Despite these developmental differences in key periods of plasticity, the rodent outcome data appears similar to a number of studies that also report programming of altered energy balance following early life nutritional perturbations in large animal models including sheep, swine and non-human primate (NHP). In the sheep, both twinning and periconceptional undernutrition are associated with epigenetic changes in fetal hypothalamic POMC and glucocorticoid receptor genes, potentially resulting in altered energy balance regulation in the offspring [54]. A recent study in sheep reported no effect of IUGR on early postnatal hypothalamic energy balance gene expression; this was associated with major sex differences in adiposity and leptinemia [55]. These IUGR offspring were suckled by overnourished mothers that may explain the lack of hypothalamic phenotype in this model. As with the rodent studies, programming effects related to undernutrition can be sexually dimorphic. As an example, male offspring from undernourished ewes develop increased adiposity which is not observed in females [56]. It is possible that these effects are a consequence of decreased central insulin action resulting in lower POMC expression thereby reducing food intake [48] or estrogen-mediated regulation of leptin and insulin resulting in compensatory mechanisms in females to curtail the effects of undernutrition. In a swine model of maternal energy restriction, differences in hypothalamic gene expression at birth and higher growth and adiposity in adulthood suggested a programming effect in offspring towards a positive energy balance, possibly due to overexposure to endogenous stress-induced glucocorticoids [57]. Moreover these effects were sex specific with only females affected.

In addition to the extensive work on the central pathways involved in energy balance and thermogenesis, there are now also reports related to altered maternal nutrition and skeletal muscle response to changes in energy balance. There is evidence that a maternal LP diet and postnatal HF nutrition may increase the risk for T2DM by decreasing skeletal muscle oxidative respiration via increased Sirtuin (Sirt)-3 and possibly by decreased amounts of the active form of succinate dehydrogenase enzyme. Undernutrition during pregnancy can also lead to dysfunctional cardiac muscle respiration in adult offspring [58]. Maternal obesity can also have adverse effects on muscle development [59] with a maternal HFD significantly decreasing muscle myogenic differentiation 1 (MYOD1) and glucose transporter type 4 (GLUT4) mRNA expression in male rat offspring which is normalized by maternal exercise [60]. Maternal obesity can also lead to alterations in regulators of mitochondrial function in skeletal muscle of offspring (including peroxisome proliferator-activated receptor gamma coactivator (PGC1)α, PGC1β) which is paralleled by reduced energy expenditure and impaired fat utilisation as assessed by indirect calorimetry [61].

Much of the early work in the developmental programming field focused on models of maternal undernutrition to induce varying degrees of fetal growth restriction. However, there are now a large number of models detailing the effects of a maternal/neonatal obesogenic environment on altered energy balance in offspring. These approaches are detailed in a recent review by Williams *et al.* [62]. A number of studies have now shown that a maternal obesogenic environment plays a critical role in programming of hypothalamic pathways that regulate feeding and energy balance [53]. In the rodent, overfeeding in the early postnatal period increases the orexigenic peptide NPY and AgRP in the arcuate nucleus of the hypothalamus [53] and offspring of mothers fed a HFD have reduced sensitivity to the anorectic effects of leptin [63,64]. A HFD during pregnancy can stimulate the proliferation of neuroepithelial and neuronal precursor cells of the embryonic hypothalamic third ventricle and stimulates the proliferation and differentiation of neurons and their migration toward hypothalamic areas where ultimately a greater proportion of the new neurons expressed orexigenic peptides. This increase in neurogenesis, closely associated with increased blood lipids, may play a role in producing the long-term behavioural and physiological changes observed in offspring after weaning, including an increase in food intake, preference for fat, hyperlipidemia, and higher body weight [65]. Rats fed a highly palatable cafeteria diet for 2 weeks show impaired sensory-specific satiety following consumption of a high calorie solution and these deficits remain even following the withdrawal of the cafeteria foods [66]. Further, a cafeteria diet can lead to neuroadaptive responses in brain circuits underlying reward with unrestrained consumption of palatable food shown to increase the reinforcing value of food and weaken inhibitory control [66,67]. In the context of developmental programming, it has been shown that perinatal exposure to high-fat, high-sugar diets results in altered development of the central reward system, resulting in increased fat intake and altered response of the reward system to excessive junk-food intake in offspring in postnatal life [68]. Maternal gestational diabetes (GDM) has also been shown to program changes in the hypothalamic neural network controlling appetite in offspring. Experimentally induced diabetes leads to offspring with increased glucose and insulin concentrations, hyperphagia and increased body weight. Offspring of GDM mothers also exhibit structural alterations that affect the density of AgRP and MSH fibers in the paraventricular nucleus with impaired leptin sensitivity [69]. Plagemann *et al.* have reported that offspring of GDM mothers have increased numbers of NPY and galanin containing neurons thus further highlighting that maternal diabetes leads to “malprogramming” of hypothalamic neuropeptidergic neurons in offspring [70]. Importantly, restoration of these neural circuits can be achieved by normalising gestational hyperglycemia [71].

As with the undernutrition models, rodent models are the most widely used in the investigation of programming effects arising from a maternal obesogenic environment. However, a range of large animal models have also been utilised in this area. Maternal obesity in the sheep increases fatty acid synthesis, upregulates nutrient transporters, and increases adiposity in adult male offspring after a feeding challenge [72]. Lambs born to obese sheep have elevated cortisol concentrations and this may play a role in disrupting the normal peak of leptin thereby predisposing them to increased appetite and weight gain in later life [73]. Further, maternal obesity in this model appears to lead to altered programming of the leptin surge that is transmitted to the F2 generation [74] with similar effects reported in a piglet model of maternal obesity [75,76,77]. In the NHP, chronic consumption of a HFD during pregnancy, independent of maternal obesity and diabetes, leads to widespread activation of proinflammatory cytokines that may alter the development of the melanocortin system. The abnormalities observed in the fetal POMC system, if maintained into the postnatal period, may therefore impact several systems, including body weight homeostasis, stress responses, and cardiovascular function.

## 4. Epigenetic Mechanisms

An alternative mechanism by which environmental factors at critical periods of development could have long-term phenotypic consequences may involve epigenetic modifications (including DNA methylation, histone modifications and microRNA (miRNA)). Epigenetic processes are now widely viewed as a leading mechanism to explain the lifelong persistence of such developmental programming of energy balance [78,79]. As epigenetic regulation during development undergoes dynamic changes, the epigenome displays a labile nature, which allows it to respond and adapt to environmental stressors, including suboptimal nutrition [80]. A number of studies have therefore focussed on the epigenetic contributions to disease manifestation arising as a consequence of adverse early life events [81,82,83]. In the setting of altered early life nutrition, organisms can fine tune gene expression to achieve environmental adaptation via epigenetic alterations of histone markers of gene accessibility [84]. Experimental models of programming have now shown that a range of challenges during pregnancy or early neonatal life result in changes in promoter methylation which directly or indirectly affect gene expression in pathways associated with a range of processes related to energy balance [85,86,87,88,89]. Mechanisms underlying epigenetic modification of tissue function resulting in a predisposition to altered programming of leptin and insulin signalling are discussed by Holness *et al.* [90]. Leptin has a 3-kb promoter region embedded within a CpG island and contains many putative binding sites for known transcription factors including a glucocorticoid response element. It has been shown that leptin’s promoter is subject to epigenetic programming, and leptin’s expression can be modulated by DNA methylation [91,92,93]. Methylation of a CpG island in the leptin promoter plays an important role in leptin expression during pre-adipocyte differentiation [92,93]. Protective effects of leptin during the suckling period against later obesity may be associated with changes in promoter methylation of the hypothalamic POMC gene [32]. In addition, activation of the leptin receptor also induces expression of suppressor of cytokine signaling-3 (SOCS-3). This protein inhibits further leptin signal transduction and also inhibits signalling by the insulin receptor. Altered SOCS-3 methylation may therefore have effects on the leptin-insulin feedback loop (the adipoinsular axis) and adversely impact developmental programming [90]. Methylation of a proximal region of the leptin promoter constitutes a significant determinant of leptin expression in human adult tissues [94] and variation in DNA methylation of the leptin promoter in animals models varies with the degree of obesity [95,96] and the epigenetic regulation of leptin signalling pathways can be manipulated by nutrients and food compounds as detailed recently [97]. In addition, leptin treatment during the neonatal period in the setting of maternal undernutrition can reverse the adverse programming effects in offspring [34,35,37]. Further, leptin treatment during the neonatal period has been shown to promote epigenetic modifications in the POMC promoter, which may confer protection from an obesogenic environment in adulthood [32].

In addition to leptin, a further candidate re epigenetic regulation is that of the fat mass and obesity-associated (FTO) gene [98,99,100]. The potential programming effects of FTO in the developmental of obesity has recently been reviewed by Sebert and colleagues [98]. Both sheep and rat models (utilising both under- and overnutrition) have shown that the FTO gene may be a key target for nutritional programming. As an example, altered FTO methylation and gene regulation may provide a link between obesity-associated leptin resistance following IUGR and rapid postnatal weight gain [101]. However, as with many methylation data, although strong associations between FTO and BMI are consistently replicated in humans, the precise biological mechanisms by which FTO regulates weight gain remains unclear (e.g., does RNA demethylation impact on the control of energy balance). In addition, different nutritional perturbation lead to differential effects on FTO regulation e.g., placental expression of the obesity-associated gene FTO is reduced by fetal growth restriction but not by macrosomia in rats and humans [102]. Interestingly, it has been shown that the beneficial effects of early short-term exercise in offspring of obese mothers are accompanied by alterations in the hypothalamic expression of appetite regulators and FTO gene [103]. There are also data related to specific vulnerability genes—owing to single nucleotide polymorphisms (SNPs)—which place the individual at risk for obesity when the affected genes are expressed or silenced [104]. As one example, several longitudinal cohorts of children assessed genetic contributions to childhood obesity and confirmed a strong association with a polymorphism in the FTO gene. Expression of the SNP-related allele has been correlated with increased BMI, adiposity, circulating leptin, and energy intake; impaired control of energy balance; and satiety [105,106,107].

One of the molecular phenotypes associated with IUGR in the rat is decreased expression of pancreatic and duodenal homeobox factor-1 (PDX1), a key transcription factor regulating pancreatic development. Recently, reduced PDX1 activity was associated with alterations in histone modifications [108]. It addition to leptin, it has been shown that the glucagon-like peptide-1 analog Exendin-4 increases histone acetylase activity and reverses epigenetic modifications that silence PDX1 in the IUGR rat [109]. Further, in line with lifestyle modifications preventing mitochondrial alterations and metabolic disorders, exercise has also been shown to change DNA methylation of the promoter of PGC1α to favour gene expression responsible for mitochondrial biogenesis and function [110].

In addition to maternal dietary restriction models, a number of reports now suggest that maternal obesity and early life overnutrition can elicit epigenetic changes in offspring. For example, overnutrition during the suckling period can lead to epigenetic modifications in key genes involved in the insulin signaling pathway in skeletal muscle and lead to later development of insulin resistance [111]. Marco *et al.* showed that a maternal HFD induced hypermethylation of the hypothalamic POMC promoter and obesity in post-weaning rats [112]. A maternal HFD can also alter methylation and gene expression of dopamine and opioid-related genes thus is a further potential mechanism for programming of appetite and preference for energy dense foods in postnatal life [113].

In addition to programming effects on DNA methylation, there is also evidence, albeit limited, for a role of the early life nutritional environment in alterations in miRNA profiles and histone modifications. miRNAs are able to control gene expression at a post-transcriptional level, thus representing an important class of regulatory molecules, and are also directly connected to the epigenetic machinery through a regulatory loop [114]. miRNAs play an essential role in the maintenance of insulin signaling and glucose homeostasis and dysregulation of miRNAs (e.g., miR-103, miR-107, miR-802 and the let-7 family) is associated with features of the metabolic syndrome, obesity, and type 2 diabetes in a number of experimental models [115,116,117,118]. An essential role for miRNAs has also recently been described for the maintenance and differentiation of brown adipocytes [119]. Experimentally, maternal undernutrition around the time of conception can induce changes in the expression of miRNAs in offspring which appears to play a role in the development of insulin resistance in later life [120]. Periconceptional or preimplantation undernutrition in the sheep leads to alterations in a number of miRNAs that relate to insulin signalling and gluconeogenesis [121]. Similar effects have been reported in a sheep model of maternal obesity with altered regulation of miRNAs involved in insulin signalling in offspring—these effects being ablated with a period of dietary restriction in the periconceptional period [122].

A number of histone-modifying enzymes have also been identified, including histone lysine, arginine methyltransferases and different classes of histone deacetylases (HDACs) with altered expression linked to a range of metabolic disorders. Several reports have now described the direct effects of dietary methyl group inadequacy on the modification of histone proteins [123]. Pogribny *et al.* demonstrated that feeding rats a methyl donor-deficient diet resulted in loss of histone H3K9 and H4K20 methylation and a role for folate in enzymatic degradation of histones [124]. However, most work to date on histones has examined effects related to carcinogenesis with little known in the context of alterations in systems regulating energy balance in the setting of developmental programming. At a mechanistic level, studies in humans linking epigenetic change to metabolic disease risk remain limited although there is evidence for inheritance of tissue specific DNA methylation patterns [125]. Aims currently pursued are the early identification of epigenetic biomarkers concerned in individual’s disease susceptibility and the description of protocols for tailored dietary treatments/advice to counterbalance adverse epigenomic events. These approaches will allow diagnosis and prognosis implementation and facilitate therapeutic strategies in a personalised “epigenomically modelled” manner to combat obesity related disorders of energy balance [126]. However, it needs to be noted that most work to date is associative in nature and the extent to which epigenetic modifications may mediate the effects of developmental programming of altered energy balance remain unclear. For example, measuring DNA methylation in blood may provide little linkage with phenotype and therefore cannot be easily justified on a functional basis [24]. Furthermore, a recent review by Waterland raised a number of questions including: in what cells/tissues is epigenetic regulation most important for energy balance? Does developmental programming of human body weight regulation occur via epigenetic mechanisms? Do epigenetic mechanisms have a greater impact on food intake or energy expenditure [78]? So far, these mechanisms include permanent structural changes in organs arising from suboptimal levels of important factors during a critical developmental period, changes in gene expression caused by epigenetic modifications and permanent changes in cellular ageing.

## 5. Brown Adipose Tissue (BAT)

In experimental models, alterations in maternal nutrition leads to altered adipogenesis in offspring including promoting an increased number of adipocyte precursor cells and an increase in expression of genes involved in both white and brown adipogenesis [24,127]. In particular, recent attention has turned to the role of BAT. Until recently, BAT was primarily viewed as the thermogenic organ for maintenance of core body temperature in small mammals with the role in humans restricted to the newborn period. Recent reports of BAT in adult humans have resulted in a renewed interest in BAT and it is now postulated to play a vital role in the regulation of energy balance and obesity in humans [24,128]. Uncoupling protein 1 (UCP1) drives non-shivering thermogenesis within BAT and, in the context of developmental programming, fetal and neonatal expression of UCP1 in BAT is regulated by maternal nutrition during gestation [129]. In the sheep, BAT genes in pericardial adipose tissue of newborns are downregulated by maternal nutrient restriction in late gestation [130], thus potentially placing these offspring at increased risk of hypothermia after birth. As with most other outcomes related to developmental programming, the magnitude and direction of the offspring response is dependent upon the timing and duration of the nutritional perturbation [24]. As an example, suboptimal maternal nutrition during early- to mid-gestation in the sheep can increase UCP1 expression, while a similar nutritional challenge in late gestation can reduce UCP1 expression in a fat depot specific manner [131]. In the rat, excess weight gain during the early postnatal period (via litter size reduction) is associated with permanent reprogramming of BAT adaptive thermogenesis (including persistent changes in UCP1) and sympathetic outflow (decreased beta 3-adrenergic receptor expression and diminished response to the sympathetic receptor agonist isoproterenol) [132]. However, the physiological roles of human BAT remain highly complex, and may exert potential health impacts beyond thermogenesis and body weight regulation with interactions with core physiological processes including glucose homeostasis, cachexia, physical activity, bone structure, sleep, and circadian rhythms [128].

## 6. Conclusions

The exquisite system for regulation of energy balance is established once in each individual’s life. In addition to genetics, environmental influences during critical periods of developmental plasticity determine the outcome of this process, with lasting consequences for body weight regulation [78]. A number of epidemiological and clinical observations and a wide range of animal models across a range of species have clearly highlighted the importance of the early life environment in programming of many aspects of physiology and behaviour including metabolism, body weight set point and energy balance regulation. Further, more recent studies have illustrated how the finely tuned long-term control of energy intake and of energy expenditure are both developmentally plastic and susceptible to environmentally-induced change that may persist with that individual throughout their adult life, invariably rendering them more susceptible to greater adipose tissue deposition [133]. A wide range of nutritional interventions in pregnancy and lactation, including both undernutrition and maternal obesity, can lead to range of metabolic disorders in offspring, which are mediated in part by epigenetic processes encompassing the chromatin information encrypted by DNA methylation patterns, histone covalent modifications and miRNA [126].

Offspring from both undernourished dams and those dams fed an obesogenic diet present with hypothalamic reprogramming with a number of alterations that appear similar across the range of experimental animal models: altered neurogenesis and neuronal functionality, disorganization of feeding pathways, modified glucose sensing, and leptin/insulin resistance [50]. Overall, these alterations may account for the long-lasting dysregulation of energy balance and obesity following adverse early life events. An increasing focus has been on epigenetic readouts but to date these remain largely associative and functional roles of observed changes are not yet established. However, perinatal epigenetic analysis may have utility in identifying individual vulnerability to later obesity and metabolic disease e.g., epigenetic gene promoter methylation at birth and later childhood adiposity [134]. However, there are still many key questions to be answered: How responsive is the system re interventions and what are the critical windows of development at which strategies should be targeted; how many generations does it take to reverse epigenetic imprinting and can reliable markers be developed for disease prediction? [135]. Evidence to date suggests that a “one size fits all” approach is problematic with some evidence that leptin or methyl donor treatment, for example, in the setting of normal pregnancies may indeed elicit adverse outcomes in offspring [36,136].

Given their importance in orchestrating and stabilizing cellular differentiation, epigenetic mechanisms must play a central role in maintaining the various physiological set points that act to promote obesity in our current obesogenic environment [78]. Dietary components may reshape the genome *in utero* and epigenetic changes induced during early life may permanently alter the phenotype in the adult organism [79]. As such, a better understanding of the epigenetic basis of developmental programming and how these effects may be transmitted across generations is essential for the implementation of initiatives aimed at curbing the current obesity and diabetes crisis. Further, many studies to date have failed to recognise the importance of sex-specific effects in programming and these differences need to be accounted for in future studies as they aid in the mechanistic understanding of how phenotypes evolve. Adopting a life course perspective allows identification of phenotype and markers of risk earlier [137], with the possibility of nutritional and other lifestyle interventions that have obvious implications for prevention of non-communicable diseases, particularly in populations undergoing nutritional transition.
